# The Impact of Cognitive Load and Mathematical Proficiency on the SNARC Effect

**DOI:** 10.3390/bs16060878

**Published:** 2026-06-01

**Authors:** Xinxin Zhang, Bihua Cao, Fuhong Li

**Affiliations:** 1 School of Psychology, Jiangxi Normal University, Nanchang 330022, China; zhxx918@aliyun.com; 2 Psychological Counseling Center, Yichun University, Yichun 336000, China; 3 Institute of Brain and Psychological Sciences, Sichuan Normal University, Chengdu 610066, China; caobihua@sicnu.edu.cn

**Keywords:** SNARC effect, cognitive control, mathematical experience

## Abstract

The spatial–numerical association of response codes (SNARC) effect refers to the phenomenon in which responses to small numbers are faster when the left hand is used, and responses to large numbers are faster when the right hand is used. Existing research has shown that cognitive load, such as memory load, is crucial in forming the SNARC effect, and the SNARC effect is smaller for math-skilled individuals than for those with low mathematical skills. However, the underlying mechanisms of how cognitive load and mathematical proficiency affect the SNARC effect remain unclear. This study employed a one-back number comparison task in which participants were asked to compare the current number with the N-1 number under a low or high cognitive load. The results showed that participants with low mathematical skills exhibited the SNARC effect under both cognitive loads. In contrast, participants with high mathematical skills showed the SNARC effect only under the high-load condition. These findings suggest that mathematical proficiency might be beneficial for individuals in resolving conflicts in the spatial–numerical association, but this advantage disappears under a high cognitive load.

## 1. Introduction

Spatial–numerical association is a classic question in psychological research. Restle (1970) first proposed the mental number line model, suggesting that people represent and manipulate numerical magnitudes on an imaginary number line when performing numerical tasks. Dehaene et al. (1993) adopted the mental number line model to explain the connection between numerical magnitude and the spatial position of the responding hand. It is widely demonstrated that responses are faster and more accurate when the numerical magnitude corresponds to the spatial position of the response hand and slower and less accurate when they do not. This is termed the spatial–numerical association of response codes (SNARC) effect (Dehaene et al., 1993).

The size of the SNARC effect is determined by subtracting the reaction times in the spatially congruent condition (i.e., left hand to small numbers and right hand to large numbers) from the reaction times in the spatially incongruent condition (i.e., left hand to large numbers and right hand to small numbers). Many studies have shown that various components of cognitive control, such as working memory, task switching, inhibitory control, and conflict adaptation, play crucial roles in modulating the size of the SNARC effect (Basso Moro et al., 2018; Georges et al., 2018; Pfister et al., 2013; Zhang et al., 2021). Based on the cognitive control perspective, it has been suggested that the size of the SNARC effect is determined by the cognitive control involved in resolving stimulus–response conflicts (Zhang et al., 2022, 2026).

As the level of cognitive control varies with memory load, changes in memory load can alter the size of the SNARC effect. The impact of memory load on the SNARC effect depends on the task and the type of memory involved. In magnitude comparison tasks, the SNARC effect disappears under a high spatial working memory load (Herrera et al., 2008), whereas in parity judgment tasks, it disappears under a high verbal working memory load (van Dijck et al., 2009). When memory load increases from zero-back to one-back, the SNARC effect decreases in parity judgment tasks but increases significantly in magnitude comparison tasks (Deng et al., 2017).

The size of the SNARC effect is also influenced by mathematical proficiency. Previous studies have found significant differences in the SNARC effect between individuals with high and low mathematical skills and demonstrated that highly math-skilled individuals exhibited a weaker SNARC effect than individuals with lower mathematical skills (Cipora et al., 2016; Dehaene et al., 1993; Fischer & Rottmann, 2005; Hoffmann et al., 2014). To explain this phenomenon, two theoretical explanations—representational theory and the inhibition account—have been proposed. The representational theory emphasizes the role of math-related domain-specific factors in the modulation of the SNARC effect, suggesting that, compared with individuals with lower mathematical skills, math-skilled individuals have more abstract numerical representations of numbers that do not automatically activate task-irrelevant spatial information (Cipora et al., 2016; Gibson & Maurer, 2016). In contrast, the inhibitory account emphasizes the domain-general cognitive factor in modulating the SNARC effect, suggesting that math-skilled individuals possess stronger inhibitory control, enabling them to better suppress interference from task-irrelevant information (Hoffmann et al., 2014; Wu et al., 2020). It is important to note that the two accounts are not mutually exclusive; they may interact in shaping the SNARC effect. Nevertheless, they generate distinct predictions regarding the effect of cognitive load.

According to the representation theory, if math-skilled individuals have flexible numerical representations, their higher numerical representation ability is unlikely to be affected by cognitive load. In other words, increasing cognitive load would not diminish the numerical ability formed by long-term mathematical experience, nor would it increase the likelihood of automatically activating task-irrelevant spatial information. Thus, the SNARC effect in the number task would not be influenced by cognitive load. Consequently, under both low and high loads, math-skilled individuals should continue to show a weaker or absent SNARC effect compared with the control group. Conversely, according to the inhibition account, math-skilled individuals may possess stronger inhibitory control (Cipora et al., 2016; Gibson & Maurer, 2016), but this advantage is constrained by limited cognitive resources. Since inhibitory control is a component of cognitive control (Hoffmann et al., 2014; Wu et al., 2020), when cognitive control resources are limited, the resources available for inhibitory control decrease. Thus, the increased cognitive load may occupy cognitive control resources, resulting in a decrease in the efficiency of inhibiting interference from the spatial mapping of the number when coding the spatial attribute of the response hand. Examining changes in the SNARC effect in math-skilled individuals under different cognitive load conditions can help clarify this theoretical controversy. It may also shed light on the underlying mechanism through which mathematical proficiency modulates the SNARC effect.

To achieve the above objectives, the present study employed a one-back number comparison task with different levels of cognitive load. The one-back task is a well-established task for inducing working memory load while keeping task structure highly similar across load conditions (Owen et al., 2005). Moreover, unlike higher-order n-back tasks (e.g., two-back or three-back) that introduce additional executive processes, such as monitoring and updating of multiple items, the one-back task primarily taxes the updating of a single item in working memory, making it more interpretable regarding the specific cognitive component being manipulated (Braver et al., 2007). We recruited two groups of participants with different levels of mathematical proficiency (i.e., college students majoring in literature or science). Each participant was asked to complete the one-back task under either the high- or low-load condition. Under high-load conditions, memory updates and continuous responses are required. Specifically, participants are sequentially presented with numbers (1–9) and must judge whether the current number is larger or smaller than the N-1 number. In this task, participants are required to continuously update their working memory. That is, after comparing the current number with the N-1 number, they must store the current number in their working memory and replace the N-1 number to serve as the new standard stimulus for number comparison in the next trial. Consequently, we refer to our high-load manipulation as a “combined cognitive load” encompassing working memory updating and executive control (van Dijck & Fias, 2011).

In contrast, in the low-load condition, participants are sequentially presented with two numbers and must judge whether the latter number is larger or smaller than the former. After the response, the next pair of numbers was presented. Under this condition, although participants needed to compare the magnitude of the current number with the N-1 number, they did not need to store the current number in working memory as the new standard stimulus for the next trial after completing the comparison; thus, there was no memory update load. Additionally, the number of key presses required by participants in this condition was reduced by half. Therefore, the cognitive load of response execution was also reduced.

We predicted that in the low-load condition, math-skilled participants might exhibit a weaker SNARC effect than participants with low levels of mathematical proficiency (Cipora et al., 2016; Hoffmann et al., 2014; Kramer et al., 2018). In the high-load condition, if the representational account were correct, the SNARC effect should not differ between high and low loads for math-skilled participants (Cipora et al., 2016; Gibson & Maurer, 2016); if the inhibition account were correct, the math-skilled participants should show a significant SNARC effect due to depleted inhibitory resources (Hoffmann et al., 2014; Wu et al., 2020).

## 2. Materials and Methods

### 2.1. Participants

G*Power 3.1 was used to estimate the required sample size. With parameters set to a medium effect size *f* = 0.25, α err prob = 0.05, and power = 0.95, the analysis indicated a minimum of 24 participants was needed for the 2 × 2 × 2 × 2 mixed design. The cControl (CON) group included 18 students who majored in language and literature, education, law, etc., and the math-skilled (MS) group included 17 students who majored in mathematics, computer science, chemistry, etc. Their ages ranged from 18 to 23 years (*M* = 19.55 years; *SD* = 1.17 years). All participants were right-handed, had normal or corrected-to-normal vision, and were paid upon completion.

### 2.2. Stimuli

Thirty pairs of numbers (1–9) were formed, with 15 ascending (e.g., 2–3) and 15 descending (e.g., 6–4). To control for distance effects, the numerical distance (the difference between two numbers) was restricted to 1 or 2. To prevent participants from using a lateralization strategy, two match trials (1–1 and 9–9) requiring a spacebar press were added. Match trials were excluded from the data analysis.

The stimuli were presented in black Times New Roman font (size 56) against a gray background on a 19-inch color monitor with a refresh rate of 75 Hz and a resolution of 1024 × 768. The viewing distance was approximately 70 cm.

### 2.3. Procedure

**Figure 1:**
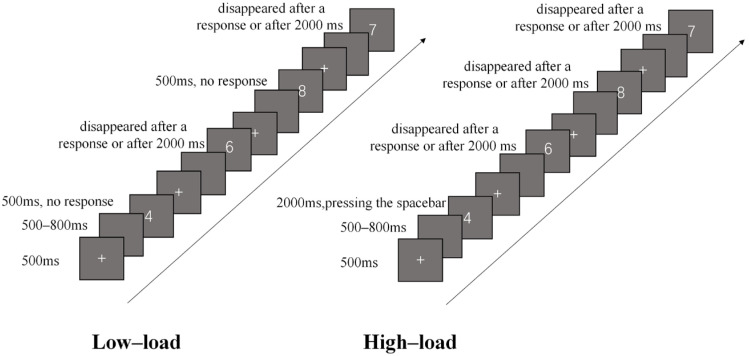
Schematic diagram of the task procedure for 1-back task in low-load condition (**left**) and 1-back task in high-load condition (**right**).

In the low-load condition, a fixation cross “+” was presented centrally for 500 ms, followed by a random blank screen for 500–800 ms. The first number appeared for 500 ms. After another fixation (500 ms) and a random blank screen (500–800 ms), a second (target) number appeared. Participants were required to judge quickly and accurately whether the second number was larger or smaller than the first (Figure 1). The target number disappeared upon response or after 2000 ms. Then, the next pair of numbers appeared. The experiment comprised 4 blocks: two blocks were SNARC-congruent (i.e., left hand for “smaller” judgment; right hand for “larger” judgment), and the other two blocks were SNARC-incongruent (i.e., left hand for “larger” judgment; right hand for “smaller” judgment). Each block comprised 128 trials, totaling 512 in the low-load condition. The block order was counterbalanced across participants. In the high-load condition, the procedure was the same as that in the low-load condition, except that participants kept the target number in their working memory (WM) after making their judgment, using it as the standard for the next comparison. The high-load condition also had 512 trials.

To counterbalance task order, half of the participants completed the low-load task first and then the high-load task; the other half completed the task in reverse order. A 3–5 min break was given between tasks.

## 3. Results

Data from participants who did not perform seriously were excluded based on the following criteria: an accuracy below 60% or reaction times outside the range of 200–2000 ms on more than 10% of trials. This led to the exclusion of two participants in the low-load condition and one participant in the high-load condition. Thus, finally, under the low-load condition, there were 16 participants in the MS group and 17 in the CON group; under the high-load condition, there were 17 participants in each group. Accuracy was analyzed for all trials. Before analyzing reaction times (RTs), error trials and trials outside three standard deviations of the mean RT were removed. The total exclusion rates were 3.6% and 3.5% for the low- and high-load tasks, respectively, with per-condition rates ranging from 2.5% to 5.2% (low load) and 2.1% to 5.4% (high load). Total exclusion did not differ between load conditions (*p* = 0.34). Repeated-measures analysis was conducted to test the SNARC effect. A significant interaction between the relative magnitude (i.e., larger or smaller than the N-1 number) and the response hand indicated a relative SNARC effect. A significant interaction between the absolute magnitude (i.e., larger or smaller than five) and the response hand indicated an absolute SNARC effect (Roth et al., 2025).

A separate 2 (group: MS, CON) × 2 (response hand: left, right) × 2 (absolute magnitude: large, small) × 2 (relative magnitude: larger, smaller) repeated- measures ANOVA was performed on the accuracy and RT data. All results were corrected using the Greenhouse–Geisser method. As this study focused on the existence of the SNARC effect, only interactions involving the response hand and absolute (or relative) magnitude are reported below.

Accuracy: No significant interaction was found between the response hand and the relative (or absolute) magnitude in the low-load task. However, in the high-load task, the interaction between the relative magnitude and the response hand was significant (*F* (1, 32) = 12.65, *p* = 0.001, and *η_p_*^2^ = 0.28). Simple effects analysis showed higher accuracy for the right-hand response to “larger” numbers (*M* = 96.5%; *SD* = 3.5%) than to “smaller” numbers (*M* = 92.4%; *SD* = 6.7%) (*p* < 0.001). For the left hand, response accuracy was higher to “smaller” numbers (*M* = 95.3%; *SD* = 4.8%) than to “larger” numbers (*M* = 94.2%; *SD* = 7.2%). The interaction between the response hand and the absolute magnitude was not significant (*p* = 0.43).

We examined potential speed–accuracy trade-offs by correlating RT differences (incongruent minus congruent) with accuracy differences across participants. No significant negative correlations were observed (all *|r|* < 0.24; *p* > 0.17), indicating that the RT findings were not compromised by a trade-off.

Reaction time (RT): In the low-load task, there was a significant three-way interaction among relative magnitude, response hand, and group (*F* (1, 31) = 4.83, *p* = 0.03, and *η_p_*^2^ = 0.14). Simple effects analysis revealed that for the CON group, the right hand responded faster to “larger” numbers (*M* = 624 ms; *SD* = 138 ms) than to “smaller” numbers (*M* = 735 ms; *SD* = 165 ms) (*p* = 0.002). The left hand responded faster to “smaller” numbers (*M* = 653 ms; *SD* = 133 ms) than to “larger” numbers (*M* = 699 ms; *SD* = 129 ms) (*p* = 0.02), indicating the presence of the SNARC effect in processing the relative magnitude of numbers. For the MS group, there was no significant difference in the left-hand RT between “larger” (*M* = 620 ms; *SD* = 149 ms) and “smaller” numbers (*M* = 654 ms; *SD* = 172 ms) (*p* = 0.23), nor the right-hand RT between “larger” (*M* = 586 ms; *SD* = 159 ms) and “smaller” numbers (*M* = 635 ms; *SD* = 169 ms) (*p* = 0.06), indicating the absence of the SNARC effect. The interaction between the response hand and absolute magnitude was not significant, indicating the absence of the SNARC effect in processing the absolute magnitude of numbers.

In the high-load task, there was a significant interaction between the relative magnitude and response hand (*F* (1, 32) = 4.49, *p* = 0.04, and *η_p_*^2^ = 0.12). Simple effects analysis indicated that the right hand responded faster to “larger” numbers (*M* = 628 ms; *SD* = 137 ms) than to “smaller” numbers (*M* = 682 ms; *SD* = 173 ms) (*p* = 0.002). For the left hand, there was no significant difference between “larger” (*M* = 672 ms; *SD* = 182 ms) and “smaller” numbers (*M* = 658 ms; *SD* = 134 ms) (*p* = 0.46). For “larger” numbers, responses were faster with the right hand (*M* = 628 ms; *SD* = 137 ms) than with the left hand (*M* = 672 ms; *SD* = 182 ms). For “smaller” numbers, responses were faster with the left hand (*M* = 658 ms; *SD* = 134 ms) than with the right hand (*M* = 682 ms; *SD* = 173 ms), indicating the presence of the SNARC effect in processing the relative magnitude of numbers. The interaction between the response hand and the absolute magnitude was not significant, indicating the absence of the SNARC effect in processing the absolute magnitude of numbers.

**Figure 2:**
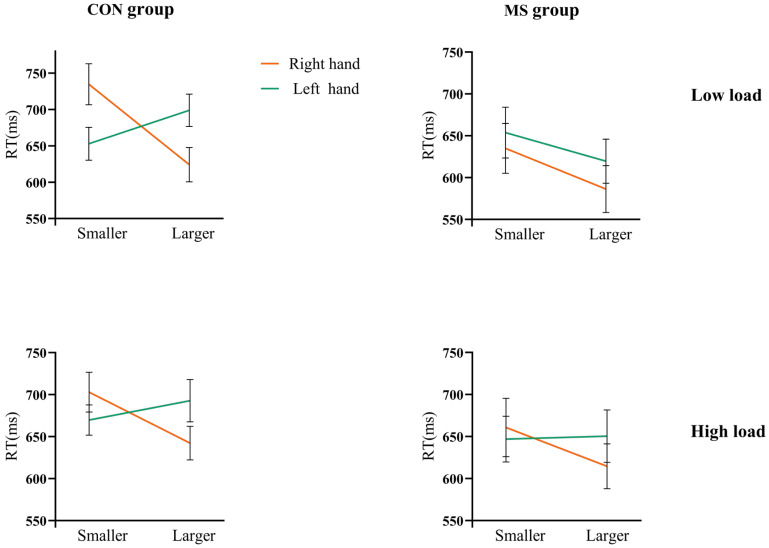
Mean reaction times of the left and right hands to different relative magnitudes in the low-load (**up**) and high-load (**bottom**) conditions for the MS group (**right**) and CON group (**left**).

As shown in Figure 2, in the low-load condition (top panels), the CON group showed a classic cross-over interaction: the left hand was faster for “smaller” numbers, and the right hand was faster for “larger” numbers, indicating a robust SNARC effect. In contrast, the MS group in the low-load condition showed roughly parallel lines (no cross-over), indicating the absence of a SNARC effect. Under high load (bottom panels), both groups exhibited a cross-over pattern: the right hand was faster for “larger” numbers, and the left hand was faster for “smaller” numbers, indicating the presence of a SNARC effect in both groups. Thus, the critical finding is that the MS group showed no SNARC effect under low load but showed a SNARC effect under high load, consistent with the inhibition account. Table 1 presents the means and standard deviations of the reaction times and accuracy for each condition by group and load.

**Figure 3:**
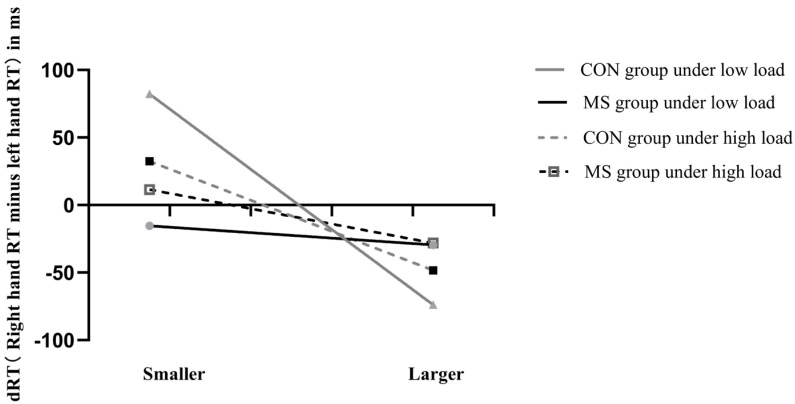
SNARC effect slopes for each group in low- and high-load tasks.

SNARC effect (slopes): Following Fias (1996), a regression analysis was performed on the mean dRTs across two magnitude levels (smaller and larger). The difference in the RT between the left and right hands (dRT) was calculated by subtracting the left-hand RT from the right-hand RT. As shown in Figure 3, for the CON group in the low-load task, the mean SNARC slope was −156.03 ms per unit magnitude (95% CI [−246.91, −65.15]), indicating a classic SNARC effect. For the MS group in the low-load task, the slope was −14.1 ms (95% CI [−119.74, 91.54]), not significantly different from zero. Under high load, the CON group showed a slope of −80.74 ms (95% CI [−153.51, −7.98]), indicating a reliable SNARC effect, while the MS group showed a slope of −47.83 ms (95% CI [−160.38, 64.71]), not significantly different from zero. The results from independent-samples *t*-tests of the size of the SNARC effect (slopes) across groups revealed that in the low-load task, the slope was bigger in the CON group than in the MS group (*t* (31) = −2.17; *p* = 0.03); no significant differences were observed among the remaining groups (*p* > 0.18).

## 4. Discussion

This study employed two types of one-back tasks to investigate the impact of mathematical proficiency and cognitive load on the SNARC effect when participants were required to judge the relative magnitude of numbers. In each trial of the low-load task, participants were sequentially presented with a pair of numbers and were required to compare the current number with the N-1 number. This numerical-processing task was similar to those used in previous studies investigating the SNARC effect in numerical relative magnitude comparison tasks (Cipora et al., 2016; Hoffmann et al., 2014; Roth et al., 2025). The primary cognitive process involved is a comparison of the relative magnitude of numbers, which does not involve working memory updating. Consistent with previous studies, although participants processed both the absolute and relative magnitudes of numbers in this task, only the relative magnitude formed an association with the spatial information of the response hand, resulting in a SNARC effect based solely on the relative magnitude. Most importantly, in the low-load task, the CON group demonstrated a SNARC effect, while the MS group did not, indicating that mathematical proficiency can influence the SNARC effect. Although the SNARC effect slope did not reach statistical significance in either load condition for the MS group, the slope showed a gradually increasing trend.

The relationship between mathematical proficiency and the SNARC effect observed in our study is consistent with previous research, further demonstrating that individuals with higher mathematical proficiency (i.e., participants in the MS group) exhibit a weaker SNARC effect than the CON group. The impact of mathematics proficiency on the SNARC effect may be (partially) moderated by several domain-general cognitive abilities and domain-specific skills. Cipora et al. (2020) claimed that the math-related domain-specific skill plays an important role in the SNARC effect. They suggested that the emergence of the SNARC effect first required automatic processing of the meaning (magnitude or ordinality) of a number. Subsequently, this information must be associated with space, a process moderated by factors such as selective attention, flexibility of representation, multiple coding of numerical–spatial associations, embodied cognition, and spatial skills. Among these, flexibility in representing numerical magnitude and accurate coding of numerical–spatial associations are math-related skills. In contrast, domain-general cognitive abilities such as selective attention, inhibitory control, and information-processing speed also play a critical role in the SNARC effect. Similarly, beyond mathematical proficiency, the two discipline-based groups may also differ in educational experience, motivation, familiarity with numerical tasks, and task-taking strategies. In the present study, we employed a magnitude comparison task, where participants judged whether the current number was larger or smaller than the preceding one. This task explicitly makes the numerical magnitude task-relevant, in contrast with the parity judgment task typically used in SNARC research.

Loading working memory reduces the efficiency of proactive control, thereby enhancing interference effects (e.g., Stroop or Simon) under high load (de Fockert et al., 2001; Lavie et al., 2004). Likewise, a growing body of empirical evidence supports the role of inhibitory control in modulating the SNARC effect. For example, Pfister et al. (2013) showed that the SNARC effect can be rapidly modulated by top-down inhibitory control, indicating that spatial–numerical associations are not obligatory but are subject to strategic inhibition. Notably, the association between inhibitory control and the size of the SNARC effect has been observed in both children and adults (Wu et al., 2020; Zhang et al., 2026).

Under low-load conditions, the smaller SNARC effect in the MS group than in the CON group was due to domain-specific math-related skills or general domain abilities. We believe that the latter is the most plausible answer to this question. That is, compared with the CON group, the smaller or even disappeared SNARC effect in the MS group might be due to a domain-general factor, but not a domain-specific factor. If the smaller SNARC effect in the MS group is attributable to domain-general factors such as more flexible representations, as proposed by Cipora et al. (2016), then it should not be affected by cognitive load because it does not alter an individual’s mathematical ability.

In contrast, general domain factors such as inhibitory ability can explain the smaller SNARC effect in the MS group. Specifically, the SNARC effect essentially reflects the conflict between number magnitude and spatial responses (Mazzocco et al., 2011; Hoffmann et al., 2014; Georges et al., 2018; Wu et al., 2020). In the number task, individuals with higher mathematical expertise allocated fewer cognitive resources to mathematical processing and devoted more cognitive resources to the inhibitory control of conflict information, resulting in a weaker SNARC effect. Inhibitory control is crucial for general mathematical learning; children with lower mathematical skills often exhibit poorer inhibitory control, leading to difficulties in switching and evaluating new strategies when processing specific tasks (Bull & Scerif, 2001). Studies in adults also indicate a positive correlation between inhibitory control and mathematical performance (Ashkenazi & Blum-Cahana, 2023; Cragg et al., 2017).

The MS group showed no SNARC effect in the low-load task but demonstrated an SNARC effect in the high-load task. Compared with the low-load task, the high-load task was primarily characterized by an increase in the memory-updating load and response execution frequency. We propose that increased demands for memory updating and response execution frequency do not impair the domain-specific expertise of the MS group. In other words, the increased demands for memory updating and response frequency do not fundamentally reduce their ability to abstractly represent numbers, nor do they enhance their spatial abilities, thereby increasing the likelihood of automatically activating spatial–numerical associations. In short, when performing the numerical magnitude comparison task, the spatial skills and abstract numerical representations involved in establishing spatial–numerical associations in the MS group did not change with the variations in cognitive load. The spatial–numerical associations formed at high and low loads may not differ significantly. According to Cipora et al. (2020), once numerical–spatial associations are established, the emergence of the SNARC effect is modulated by individuals’ inhibitory control over interference information and processing efficiency. Memory updating and response control fall within the domain of cognitive control (Braver et al., 2007). Given that overall resources for cognitive control are limited, an increase in the cognitive load for updating and response control is likely to reduce the remaining available cognitive resources. Consequently, the resources available for resolving conflicts between spatial–numerical information and response-related spatial information decrease, reducing the efficiency of inhibiting spatial–numerical interference during keypress responses. This ultimately resulted in a pronounced SNARC effect during the high-load task in the MS group.

## 5. Conclusions

Consistent with the inhibition account, compared with individuals with weaker mathematical skills, math-skilled individuals have a stronger ability to inhibit interfering information in forming number–spatial associations. This allows them to more efficiently inhibit task-irrelevant and conflicting information, facilitating the resolution of conflicts between numerical–spatial representations and the spatial position of the response hand. When cognitive load increased, the resources available for inhibition decreased, resulting in the emergence of the SNARC effect in the MS group. These results suggest that long-term experience in math learning and skill development might play a significant role in forming numerical–spatial associations, whereas cognitive control, such as inhibition, modulates the behavioral manifestation of numerical–spatial associations and the observed SNARC effect.

### Limitations

First, future research could further increase the sample size and task difficulty. Only the one-back task was used in the present study; therefore, the curve of the SNARC effect varying with the working memory load was not investigated. Future studies should extend to two-back or even higher-cognitive-load conditions. Second, compared with low-load tasks, high-load tasks involve an increase in both memory updating and response execution. Future studies should investigate the type of load that has the greatest impact on the SNARC effect in the MS group. Finally, conclusions regarding the stage at which the SNARC effect occurs remain inconclusive. Although our findings are consistent with the inhibition account, we cannot directly demonstrate that individual differences in inhibitory control mediated the observed effects. Future research should incorporate direct behavioral or neural measures of executive functions.

## Figures and Tables

**Table 1:** Means and standard deviations of reaction times and accuracy for each condition by group and load.

Load	Group	Relative Magnitude	Response Hand	M		SD		SE	
				ACC (Proportion)	RT (ms)	ACC (Proportion)	RT (ms)	ACC (Proportion)	RT (ms)
Low load	CON group	Larger	Right	0.94	624	0.07	138	0.012	24
			Left	0.93	699	0.06	129	0.01	22
		Smaller	Right	0.93	735	0.05	165	0.008	28
			Left	0.94	653	0.07	133	0.012	23
	MS group	Larger	Right	0.96	586	0.04	159	0.007	28
			Left	0.95	620	0.04	149	0.006	26
		Smaller	Right	0.94	635	0.05	169	0.009	30
			Left	0.94	654	0.05	172	0.008	30
High load	CON group	Larger	Right	0.96	642	0.04	117	0.007	20
			Left	0.94	693	0.09	147	0.016	25
		Smaller	Right	0.92	703	0.08	138	0.013	24
			Left	0.95	670	0.05	105	0.009	18
	MS group	Larger	Right	0.97	615	0.03	155	0.005	27
			Left	0.94	650	0.05	211	0.008	31
		Smaller	Right	0.93	661	0.06	203	0.01	35
			Left	0.96	647	0.04	159	0.007	27

## Data Availability

The data that support the findings of this study are available from the corresponding author upon reasonable request.
